# Negative Regulation and Protective Function of Natural Killer Cells in HIV Infection: Two Sides of a Coin

**DOI:** 10.3389/fimmu.2022.842831

**Published:** 2022-03-07

**Authors:** Yu Sun, Jie Zhou, Yongjun Jiang

**Affiliations:** National Health and Family Planning Commission (NHC) Key Laboratory of AIDS Immunology (China Medical University), National Clinical Research Center for Laboratory Medicine, The First Affiliated Hospital of China Medical University, Shenyang, China

**Keywords:** HIV, natural killer cells, HIV reservoir, negative regulation, immune receptors, CAR-NK

## Abstract

Natural killer (NK) cells play an important immunologic role, targeting tumors and virus-infected cells; however, NK cells do not impede the progression of human immunodeficiency virus (HIV) infection. In HIV infection, NK cells exhibit impaired functions and negatively regulate other immune cell responses, although NK cells can kill HIV-infected cells and thereby suppress HIV replication. Considerable recent research has emerged regarding NK cells in the areas of immune checkpoints, negative regulation, antibody-dependent cell-mediated cytotoxicity and HIV reservoirs during HIV infection; however, no overall summary of these factors is available. This review focuses on several important aspects of NK cells in relation to HIV infection, including changes in NK cell count, subpopulations, and immune checkpoints, as well as abnormalities in NK cell functions and NK cell negative regulation. The protective function of NK cells in inhibiting HIV replication to reduce the viral reservoir and approaches for enhancing NK cell functions are also summarized.

## 1 Introduction

Natural killer (NK) cells are large granular lymphocytes and the first line of defense against tumors and viral infection (1–3). NK cells include three common subpopulation groups: CD56^bright^CD16^neg/pos^ subpopulation, which primarily secrete cytokines, the CD56^dim^CD16^pos^ subpopulation, which exerts toxic effects, and the dysfunctional CD56^neg^CD16^pos^ subpopulation (1, 4). Activating receptors (NCRs, NKG2C, NKG2D, etc.) and inhibitory receptors (IKIR, NKG2A, PD-1, TIGIT, etc.) are expressed on the surface of NK cells, and their combined activity determines the cell’s function (2, 5). NK cells can initiate immune responses to target tumors and virus-infected cells by releasing perforin, Granzyme B, cytokines, and *via* Fas/Fas-L pathway (3, 6). Human immunodeficiency virus (HIV) is a retrovirus that tends to infect CD4 T cells *via* the viral envelope protein gp120 (7), inducing the depletion of infected CD4 T cells and bystander resting CD4 T cells (8–10). Ultimately, HIV infection functionally exhausts the immune system, leading to repeated infections and tumors, and ultimately, death. Research indicates that NK cells play a crucial role in anti-HIV immune responses (11) but fail to control HIV infection due to dysfunction or exertion of negative regulatory effects (12, 13). Nonetheless, NK cells are considered potentially important in the treatment of HIV infection and have even been applied to the elimination of viral reservoirs that harbor integrated, high-replicability proviruses in the host cellular DNA that cause rebound viremia when antiretroviral therapy (ART) is stopped (14, 15).

Although numerous original articles have been published regarding NK cells as related to immune-senescence, immune checkpoints, negative regulation, antibody-dependent cell-mediated cytotoxicity (ADCC), and HIV reservoirs during HIV infection, an overall summary of these studies is lacking. Therefore, this review focuses on the characteristics of NK cells during HIV infection and summarizes the prospects of immunotherapies based on NK cells as means of eliminating viral reservoirs.

## 2 Changes in NK Cell Counts and Subpopulations in HIV Infection

### 2.1 NK Cell Counts

Considerable research indicates that NK cell counts change during HIV infection. Alter et al. found that the total number of NK cells expands in acute HIV infection (16), whereas in chronic viremic HIV infection, the total number of NK cells declines (17). Recently, Wang et al. reported that during primary infection, individuals with higher numbers of NK cells exhibit lower plasma viral loads and require a longer period for CD4 T cell numbers to decline to 500 cells/μl (18). In addition, NK cell frequencies are negatively correlated with HIV DNA levels in patients during histone deacetylase inhibitor (HDACi) treatment (19, 20). Thus, the number of NK cells could serve as a predictor of HIV disease progression.

### 2.2 NK Cell Subpopulations

During acute HIV infection, CD56^dim^CD16^pos^ NK cell subpopulations are amplified, whereas CD56^neg^CD16^pos^ NK cell populations increase slightly, and CD56^bright^CD16^neg^ NK cell populations are depleted early (16). During chronic HIV infection, the percentage of CD56^neg^ NK cells increases significantly (21). This subpopulation exhibits abnormal killing of K562 cells after recombinant IL-2 stimulation (22) and can affect the CD4 to CD8 T-cell ratio after ART; the higher the CD56^neg^CD16^pos^ NK cell subpopulation proportion before ART, the lower the CD4 T cell count after ART (23). The percentage of CD56^dim^ NK cells decreases during chronic HIV infection (21, 24). In addition, normal NK cell subpopulation distributions are maintained in nonprogressive chronic HIV infection (16). Doria et al. found that early treated (ET) patients (≤6 months after infection) exhibit normal NK cell subpopulation distributions, whereas late-treated (LT) patients (>6 months after infection) exhibit a higher percentage of the CD56^neg^ subpopulation and lower percentage of the CD56^bright^ subpopulation than ET patients (25). The above data suggest that the CD56^dim^ subpopulation is upregulated in acute infection and downregulated during chronic infection, whereas the CD56^neg^ subpopulation is upregulated in both acute and chronic infection, resulting in reduced ability to suppress HIV. The changes in these subpopulations might be useful as potential biomarkers for predicting disease progression.

In addition to these classical subpopulations, changes in other subpopulations in HIV infection should be noted. Using semi-supervised machine learning approaches, Pohlmeyer et al. found that the CD11b^pos^CD57^neg^CD161^pos^Siglec-7^pos^CD56^dim^CD16^pos^ NK cell subpopulation can be used to distinguish elite controllers from viremic non-controllers among HIV-infected individuals. The number of cells of this subpopulation declines in viremic non-controllers, and *in vitro* data indicate that this subpopulation secretes more IFN-γ and CD107a compared with other CD56^dim^CD16^pos^ NK cell subpopulations in HIV infection (26). In addition, Guo et al. found that the CXCR5^pos^ NK cell subpopulation accumulates in lymph nodes and is negatively correlated with HIV DNA levels (27). Moreover, Adeniji et al. found that a decrease in the percentage of the Siglec-9^pos^CD56^dim^ NK cells subpopulation and is negatively correlated with viral load during HIV infection. This subpopulation exhibits higher cytotoxicity, expresses higher levels of activating receptors (e.g., CD16, NKp30, and CD38) and lower levels of inhibitory receptors (e.g., TIGIT and NKG2A) compared with Siglec-9^neg^CD56^dim^ NK cells during HIV infection (28). Thus, these subpopulations might play a role in spontaneous control of HIV infection and therefore prove useful in future HIV treatments.

## 3 NK Cell Dysfunction During HIV Infection

### 3.1 Inflammatory Environment Accelerates NK Cell Senescence

HIV infection manifests as a chronic inflammatory condition, and chronic inflammation is a potential driver of immune-senescence (29). Desdín-Micó et al. recently confirmed that inflammation induces distal tissue senescence in Tfam^fl/fl^ Cd4^Cre^ mice (30). Reactive oxygen species (ROS) and telomere length are key factors in determining cellular longevity (31, 32), and Soares et al. reported that NK cells produce increased levels of ROS and exhibit shortened telomere length in individuals with chronic HIV infection (33). In addition, Campos et al. reported an increased percentage of CD56^neg^CD16^pos^ NK cells in healthy elderly individuals and lower levels of Granzyme expression compared with healthy young individuals (4). This pattern of CD56^neg^CD16^pos^ NK cell expansion and dysfunction is common in HIV-infected individuals and could be indicative of immuno-senescence.

### 3.2 Surface Receptor Imbalance Weakens NK Cell Functions

In NK cells, surface receptors play an essential role in regulating homeostatic function. HIV-infected individuals exhibit an imbalance between inhibitory and activating surface receptors of NK cells, leading to NK cell dysfunction. Inhibitory receptors are generally upregulated in HIV-infected individuals, whereas activating receptors are downregulated.

#### 3.2.1 T-Cell Immunoreceptor With Ig and Immunoreceptor Tyrosine-Based Inhibition Motif (ITIM) Domains (TIGIT) and DNAM-1(CD226)

Expression of TIGIT, an inhibitory receptor and classical immune checkpoint, is elevated in chronic infectious diseases or tumors and related to NK cell dysfunction (34, 35). Zhang et al. found that an increase in the number of NK cells expressing TIGIT leads to higher HIV load, and TIGIT^pos^ NK cells exhibit less-effective killing than TIGIT^neg^ NK cells in HIV-infected individuals (36). In contrast to TIGIT, CD226 is an activating receptor that competes with TIGIT for the ligand, CD155, and targets HIV-infected CD4 T cells expressing the CD155 ligand (36–38). Expression of CD226 is upregulated on NK cells during HIV infection (39), and Yin et al. reported that TIGIT is specifically expressed on CD226^pos^ NK cells during HIV infection (38). It is possible that TIGIT upregulation offsets the activation of CD226 on NK cells. Although TIGIT blockade enhances NK cell responses (35, 38), Vendrame et al. demonstrated that TIGIT blockade does not enhance NK cell–mediated killing of HIV-infected host cells, whereas TIGIT co-expression with multiple activation markers (CD2, 2B4, CD226, NTB-A) enhances the functional response of NK cells to tumors, cytokine activation, and virus-infected cells (40). Further studies of the function of TIGIT on NK cells during HIV infection are warranted.

#### 3.2.2 Programmed Cell Death 1 (PD-1)

Previous studies demonstrated that PD-1 expression is associated with disease progression in individuals with various tumors, and blockade of the immune checkpoint molecule PD-1/PD-L1 axis was shown to exert anti-tumor effects (41, 42). Another study reported that the proportion of NK cells expressing PD-1 increases during HIV infection (43). Porichis et al. found that combined blockade of PD-1 and IL-10 enhances the killing capacity of NK cells in HIV infection and enhances HIV-specific auxiliary CD4 T cell functions, such as induction of IL-2 and IL-12 secretion (44). IL-2 also enhances HIV-specific NK cell–mediated ADCC (45).

#### 3.2.3 CD300a

CD300a transmits inhibitory signals *via* ITIMs (46). Vitallé et al. found that CD300a is overexpressed on immature CD56^dim^ NK cells in HIV-infected individuals, and NK cell ADCC is significantly diminished due to CD300a expression; cross-linking of CD300a inhibits the CD56^bright^ NK cell subpopulation more than other subpopulations (47).

#### 3.2.4 T-Cell Immunoglobulin Mucin Domain Molecule 3 (Tim-3)

Expression of the inhibitory receptor Tim-3 (48) is not upregulated in HIV-infected individuals. Yu et al. reported reduced Tim-3 expression on NK cells in HIV-infected individuals (49), and de Kivit et al. reported reduced Tim-3 expression on NK cells in HIV immunologic non-responders compared with immunologic responders. Low Tim-3 expression is correlated with low CD4 T cell counts (50). Kared et al. reported that Tim-3 expression is negatively correlated with levels of IFN-γ secreted by NK cells after ART (51). Tim-3^pos^ NK cells suppress NF-κB and ERK phosphorylation to reduce IFN-γ production, and Tim-3 blockade induces NK cells to secrete CD107a (49). Plasma levels of the Tim-3 ligand, Gal-9, significantly increase during primary HIV infection, and soluble Gal-9 triggers the downregulation of Tim-3 expression (52).

#### 3.2.5 NKG2A

The NKG2A receptor inhibits NK cell function. During HIV infection, expression of NKG2A on CD56^dim^ NK cells increases and the percentage of NKG2A^pos^CD56^dim^ NK cells is negatively correlated with CD4 T cell count, in addition, NKG2A expression is further increased on CD56^dim^ NK cells in AIDS (53). NKG2A expression on CD56^neg^ NK cells decreases in HIV infection (53, 54), and the degranulation function of NKG2A^neg^CD56^neg^ NK cells is impaired compared with NKG2A^pos^CD56^neg^ NK cells (54).

#### 3.2.6 Natural Cytotoxicity Receptors (NCRs)

NCRs are activating receptors and include NKp30, NKp44, and NKp46. De Maria et al. reported that in HIV-infected individuals, NK cells expressing low levels of NKp30, NKp44, and NKp46, exhibit decreased cytotoxicity against tumor cells (55). Kulkarni et al. found that HIV-infected individuals with higher NKp30 expression have a lower viral set point (56). The mechanism leading to decreased NKp30 expression could involve the significantly higher plasma levels of transforming growth factor (TGF)-β in HIV-infected individuals (57, 58). Li et al. showed that NKp30 is a fungicidal receptor for NK cells; loss of NKp30 expression results in defective cytotoxicity against fungi and impaired perforin release (59). Decreased expression of NCRs diminishes NK cell activity against harmful microorganisms.

#### 3.2.7 NK Group 2D (NKG2D)

NKG2D is an activating receptor that binds to the surface ligand NKG2DL to mediate the killing function of NK cells. Numerous studies have reported a general decrease in NKG2D expression in HIV-infected individuals (21, 60). In addition, NKG2D expression is negatively correlated with viral set point (56), and the capacity of NK cells to kill target cells decreases with decreased NKG2D (61).

The above results indicate that inhibitory receptors (TIGIT, PD-1, CD300a, NKG2A) are generally upregulated on NK cells, whereas activating receptors (NCR, NKG2D) are downregulated. Inhibitory receptors in particular might be co-expressed with activating receptors in HIV infection, thereby offsetting the function of the activating receptors. As a result, NK cell function is inhibited and the antiviral effect is impaired. Inhibitory or activating receptors might therefore prove useful as targets in HIV immunotherapy.

### 3.3 Viral Proteins and Other Factors Impair NK Cell Function

Aside from an imbalance between inhibitory and activating receptors, NK cell cytotoxicity is also affected by a series of other factors, including the inflammatory environment and levels of certain proteins in HIV-infected CD4 T cells.

#### 3.3.1 HIV Viral Protein U (Vpu) and HIV-Negative Factor (Nef) Mediate HIV Immune Escape

Intercellular adhesion molecule-1 (ICAM-1) is a glycoprotein that promotes cell aggregation and inflammatory responses in HIV infection (62). ICAM-1 is reportedly correlated with NK cell–mediated killing ability (63). Tremblay-McLean et al. reported that ICAM-1 expression on CD4 T cells is increased in HIV infection (64). However, the viral protein Vpu reduces both intercellular and surface expression of ICAM-1, which prevents NK cells from killing infected CD4 T cells (65). Nef is an auxiliary protein of HIV that mediates viral persistence by modifying the local environment around infected cells (66). Nef downregulates HLA-A and HLA-B expression by accelerating surface endocytosis to escape cytotoxic T lymphocyte killing (67, 68). Conversely, Nef maintains HLA-C and HLA-E expression, which inhibits the killing activity of NK cells (69). A recent study reported that HIV Vpu mediates the downregulation of HLA-C expression (70), which could induce resistance to NK cells with HLA-C–specifically activated killer cell immunoglobulin-like receptors (71).

#### 3.3.2 Matrix Metalloproteinases (MMPs) Drive NK Cell Exhaustion

MMPs hydrolyze multiple constituents of the extracellular matrix to regulate various biological processes and diseases (72). Previous studies reported that MMP levels are increased in HIV-infected CD4 T cells and that MMPs induce the release of NKG2DL into the peripheral circulation, resulting in decreased expression of NKG2D on NK cells and a decrease in the killing ability of NK cells (73, 74).

#### 3.3.3 TGF-β and Interferon-Gamma-Inducible Protein (IP)-10 Negatively Regulate NK Cells

TGF-β and IP-10 play key roles in controlling immune responses and regulating the activity of immune cells (75, 76). Plasma levels of TGF-β are significantly increased in HIV-infected individuals (57, 77). Majumder et al. reported that increased TGF-β production by Vpr+ infected peripheral blood mononuclear cells leads to impaired ability of NK cells to lyse Vpr+ infected target cells (78). Plasma levels of IP-10 are markedly increased in HIV infection and positively associated with disease progression (79–81); increased IP-10 levels lead to decreased secretion of IFN-γ and CD107a expression by NK cells (79). IP-10 blockade could therefore be a new strategy for controlling HIV (79). In addition to the various aspects mentioned above, NK cell functions might also be affected by many other as yet unknown factors during HIV infection; thus, further study is needed. Reducing the expression of certain viral proteins and inhibiting certain elevated expression of cytokines in plasma are potential options for restoring the function of NK cells to suppress HIV infection.

## 4 NK Cells Play a Negative Regulatory Role in HIV Infection

NK cells exert multiple biological functions. In addition to anti-tumor and antiviral functions, NK cells also have regulatory functions in the maintenance of immune homeostasis. Waggoner reported that NK cells acted as rheostats in a mouse model of lymphocytic choriomeningitis virus (LCMV, a model of human HIV) infection. During high-dose LCMV infection, wild-type mice exhibited an increased survival ratio, less-intense immuno-pathogenesis, and higher viral titer compared with NK cell–depleted mice. At medium doses of LCMV, infected wild-type mice exhibited higher viral titer, fewer antiviral T cells, and lower survival ratio compared with NK cell–depleted mice (82). The regulatory functions of NK cells inhibit immuno-pathogenesis in LCMV infection but enable persistent viral infection. Lang et al. also demonstrated that NK cells expressing higher levels of NKG2D inhibit the proliferation of CD8 T cells and IFN-γ secretion during LCMV infection (83).

Other studies have found that peripheral blood from healthy human controls contains a small amount of regulatory NK (NK-reg) cells that secrete IL-10 and inhibit the proliferation of antigen-specific CD4 T cells (84). In chronic Hepatitis B Virus infection, NK-reg cells secrete more IL-10, which then suppresses the proliferation of CD4 and CD8 T cells and the secretion of IFN-γ (85). Moreover, tumor-infiltrating NK-reg cells express high levels of CD73, which promotes the production of TGF-β and IL-10 *via* the STAT3 signaling pathway. CD73^pos^ NK-reg cells also inhibit the activation of CD4 T cells (86). Recent research indicates that NK cells also play a negative regulatory role in HIV. Jiang et al. reported that the proportion of TGF-β^pos^ or IL-10^pos^ NK cells is increased in HIV infection; *in vitro* experiments indicated that recombinant IL-10 and TGF-β suppress NK cell function (13). Ma et al. further demonstrated that both the percentage and absolute number of CD56^neg^CD16^pos^ NK cells are higher in HIV-infected individuals and that CD56^neg^CD16^pos^ NK cell subpopulations secrete higher levels of TGF-β and IL-10; *in vitro* experiments indicated that CD56^neg^CD16^pos^ NK cells from HIV-infected individuals suppress IFN-γ secretion by CD8 T cells. Treatment with anti–IL-10/anti–TGF-β antibodies was shown to reverse the inhibition of CD56^neg^CD16^pos^ NK cells (87). Moreover, NK cells can also kill autologous CD4 T cells, thus impairing CD4 T cell function. Luo et al. reported that NK cells from HIV-infected immunologic non-responders exhibit increased killing activity against uninfected CD4 T cells *in vitro* (88). Additionally, Chen et al. reported that NK cells tend to kill uninfected CD4 T cells due to increased ICAM-1 expression (89).

The maintenance of homeostasis is important physiologically. HIV infection leads to over-activation of the immune system and inflammatory response, potentially resulting in immuno-pathogenic effects. The regulatory functions of NK cells work to prevent pathological damage, but they simultaneously inhibit the function of immune cells, thereby impeding viral clearance. Therefore, whether the regulatory effect of NK cells plays a positive or negative role during antiviral treatment should be explored further.

## 5 NK Cells Can Still Play an Important Role in Antiviral Function

### 5.1 The Killing Effect of NK Cells

Although NK cell function is impaired during HIV infection, it still plays a key role in the antiviral immune response. Hattori et al. demonstrated that activated NK cells exert anti-HIV effects in NOD/SCID/Jak3 null mice by directly killing HIV-infected cells (90). Increased Granzyme B content and IFN-γ level in the total NK cell population have been reported in long-term non-progressing individuals (91). Alter et al. reported that CD107a expression increased and is positively correlated with the levels of NK cell–mediated target cell lysis and viral replication in viremic HIV infection (17). NK cells also exert immunomodulatory effects *via* the release of CC-chemokine. Kottilil et al. reported that CC-chemokines such as CCL5 (RANTES) and CCL3 (MIP-1α) mediate the inhibition of HIV replication; levels of these cytokines are inversely correlated with the level of plasma viremia (92). The following possible mechanisms for NK cell–mediated killing have been proposed: (i) HLA expression in HIV-infected cells decreases *via* a missing-self mechanism, leading to activation of NK cells (93–95), or (ii) changes in the HLA-I peptide repertoire during HIV infection function as an ‘innate’ mechanism that reduces the involvement of inhibitory killer cell immunoglobulin-like receptors, thereby enhancing the ability of NK cells to recognize infected cells (96).

### 5.2 ADCC

The activity of NK cells also involves ADCC, which plays a critical role in the anti-HIV immune response. It has been reported that anti-HIV IgG can be isolated from the plasma of acutely infected persons, and plasma from these patients can inhibit HIV p24 in the presence of NK cells (97). Chen et al. reported that ADCC reduces the level of viral set point in the early stages of infection, which could have great significance in disease intervention (98). ADCC is primarily mediated by NK cells *via* binding of the FcR-γIIIa (CD16) receptor with the Fc segment of antibodies bound to antigens expressed on infected cells. NK cells are activated by CD16 *via* signals associated with small adaptor proteins, such as FcϵRI-γ(FcRγ) and CD3-ζ (99). However, CD16 expression is not the only factor that determines the intensity of ADCC. FcRγ^neg^ NK cells expressing lower levels of CD16 compared with traditional NK cells in HIV-infected individuals exhibit enhanced ADCC after CD16 cross-linking (100, 101), although the mechanism remains unclear. Several approaches to increase ADCC have been reported. For example, increased IFN-α secretion significantly augments ADCC-associated lysis of HIV-infected target cells (102). In addition, NKG2D can assist CD16 to exert ADCC effect (103); adding alefacept (recombinant human LFA-3/IgG1) was shown to further enhance the preference of CD16.NK-92 cells for killing infected CD4 T cells (104). Bardhi et al. constructed a broadly neutralizing antibodies (bnAb) (defucosylated LSEVh-LS-F) that increases the affinity between the antibody’s Fc segment and CD16 on NK cells, specifically targeting two sites: the gp120 CD4 binding site and CD4-induced gp120 co-receptor binding site, thereby enhancing ADCC in a humanized mouse model (105). This effect was also observed with another bnAb targeting the gp41 region of the HIV Env protein (106).

### 5.3 Memory/Adaptive NK Cells

The discovery of memory NK cells prompted the novel proposal of directly protecting the host by exploiting the effector function of memory NK cells (107). Nikzad et al. discovered that human NK cells segregated from the BLT (bone marrow, liver, and thymus) humanized mice vaccinated with HIV-Env exhibit vaccination dependence and mediate the specific killing of HIV-Env–loaded homogenic cells *in vitro* (108). The proportion of the adaptive-like CD56^dim^ NK cell subpopulation exhibiting high expression of CD57, NKG2C, and CD2 and low expression of FcRγ is increased in HIV-infected individuals (109, 110). Several immunological and virological markers are improved in HIV-infected individuals with a high frequency of NKG2C^pos^CD57^pos^ NK cells, including lower HIV viral load, lower IP-10 and IL-6 plasma levels, and normal mature dendritic cell counts (111, 112). In addition, the adaptive NK cells contribute to the efficacy of early ART, and notably, the frequency of these cells does not decline after early ART (111). Tomescu et al. reported that during the course of bnAb immunotherapy, CD57^pos^ and NKG2C^pos^ NK cells are the primary ADCC effector subpopulations targeting HIV-infected CD4 T cells (113). Ma et al. reported that the percentage of NKG2A^neg^NKG2C^pos^ NK cells is negatively associated with plasma HIV RNA levels, and NKG2C^pos^ NK cells from HIV-infected individuals were shown to inhibit the P24 antigen to a greater extent *in vitro* than NKG2C^neg^ NK cells (114). Moreover, CD57^pos^NKG2C^pos^ NK cells exhibit higher OSBPL3 expression, which is generally linked to ADCC (115). Wang et al. recently reported that the frequency of CD94^pos^TCF7^pos^CD56^hi^ NK cells is increased in HIV-infected individuals enriched with transcripts related to lymphocyte memory. After IL-12/IL-15 combined stimulation, CD94^pos^CD56^hi^ NK cells exhibit increased IFN-γ production and degranulation against HIV-infected CD4 T cells compared with the CD56^dim^ NK cell subpopulation (116). Expansion of this subpopulation could occur secondary to the inflammation caused by loss of homeostatic type 3 innate lymphoid cells residing in the gut (116, 117). NK cells kill target cells and inhibit viral replication, thereby impeding the progression of HIV infection. NK cells exhibiting properties of memory cells were discovered in recent years. These cells are characterized by a long lifespan and rapid responsiveness to HIV-infected cells. Therefore, these properties of NK cells could be exploited in future HIV treatments.

## 6 NK Cell–Based Immune Interventions Provide a Promising Strategy to Overcome Viral Reservoirs

Although ART can control viral replication, dormant viruses cannot be eliminated, resulting in the formation of viral reservoirs. The difficulty of eliminating latent HIV reservoirs remains the greatest obstacle to curing HIV infection. NK cells play a critical role in suppressing viral reservoirs. Marras et al. reported that the frequency of IFN-γ^pos^ NK cells is inversely correlated with the size of the viral reservoir, measured as the integrated HIV DNA level in HIV individuals (118). It has also been reported that the frequencies of total NK cells are negatively correlated with HIV reservoir size measured as total HIV DNA in patients treated with HDACis (19, 20, 119). Thus, immunotherapies that enhance the function of NK cells or utilize engineered NK cells could become effective strategies for eliminating the HIV viral reservoir.

### 6.1 Strategies for Enhancing NK Cell Function

#### 6.1.1 Blocking Inhibitory Receptors for NK Cell Function

Multiple inhibitory receptors that suppress cell function are expressed on NK cells. It has been widely reported that blockade of NK cell inhibitory receptors enhances the function of the cells in inhibiting tumor immune escape, and similar observations have been reported for HIV infection. Lisovsky et al. reported that blockade of NKG2A *in vitro* increases CD56^dim^NKG2A^pos^ NK cell degranulation and secretion of IFN-γ and CCL4 against infected CD4 T cells (120). As mentioned in Section *Surface Receptor Imbalance Weakens NK Cell Functions* blockade of immune checkpoints such as Siglec-9, TIGIT, PD-1, and Tim-3 also enhance the killing activity of NK cells in HIV infection (28, 38, 44, 49). Inhibitors of NK cell inhibitory receptors might thus prove useful for promoting NK cell functions in HIV infection.

#### 6.1.2 Specific Latency Reversal Agents (LRAs) for NK Cell Function

Although LRAs are widely used in the treatment of virus reservoirs, some LRAs can be toxic and damage immune cells (121, 122). Many recent studies have explored various LRAs that enhance NK cell function. SAHA, an HDACi, reactivates latent HIV and upregulates NKG2DLs of infected cells based on ATR kinase, thus exposing infected T cells to NKG2D-mediated clearance by NK cells (123). PRO, a protein kinase C agonist, activates NK cells and markedly increases CD69 and CD107a expression (122). PRO/romidepsin combination treatment was shown to enhance NKG2D-mediated NK cell killing of P24 targets compared with single-drug treatment (121). TLR agonists also reverse HIV latency and enhance innate antiviral immunity (124). TLR-9 agonists increase NK cell degranulation, IFN-γ production, and HIV-specific inhibition (125). For effective suppression of viral reservoirs, it is necessary to choose an LRA that enhances NK cell effector activity.

#### 6.1.3 Cytokine Adjuvants for NK Cell Function

IL-18, IL-15, IL-12, and IL-2 have been recognized to activate and enhance NK cell function (126, 127). Ju et al. reported that administration of IL-21 increases the frequency of NK cells in HIV-vaccinated mice (128). Garrido et al. reported that NK cells stimulated with IL-15 recognize and eliminate cells latently infected with HIV after reactivation during LRA treatment (129). Moreover, IFN-α reduces the HIV replication *via* activation of immune effector NK cells, without inhibiting CD8 T cell function (130). Pegylated IFN-α−2a enhances the cytotoxicity of NK cells, thereby suppressing viral reservoirs (131, 132). A possible mechanism involves binding of IFN-α to the IFN-αβ receptor, which activates multiple downstream signaling pathways, such as STAT1/STAT4, to induce the secretion of IFN-γ by NK cells (133).

### 6.2 Prospects for Chimeric Antigen Receptor (CAR)-NK Cell Treatment of Viral Reservoirs

Immune cells can be engineered to express a CAR, thereby redirecting their specificity and focusing their killing capacity on a particular antigen. The basic structure of a CAR molecule includes an antigen-recognition domain comprised of an intracellular signaling domain, a transmembrane domain, an extracellular hinge domain, and a single-chain variable fragment (134). In clinical trials, Liu et al. found that CAR–NK cell therapy is effective in individuals with refractory CD19-positive cancers and does not exhibit severe toxicity (135). CAR–NK cells exhibit characteristics typical of non-engineered NK cells and are resistant to HIV infection. In addition, their use is associated with substantially fewer side effects related to toxicity compared with other treatments. Therefore, CAR–NK cells could be clinically useful for targeting and eliminating latent HIV–infected cells.

Zhen et al. (2015) constructed a triple-CAR vector comprising the CD4ζ CAR and two antiviral genes (targeting specific HIV long terminal repeat sequences and human CCR5, respectively). This triple-CAR vector was transduced into human hematopoietic stem and progenitor cells, which differentiated into effector CAR–NK cells and CAR–T cells after transplantation into γc−/− NSG mice. These CAR cells significantly suppressed HIV replication *in vivo* (136). Moreover, Lim et al. used 2,4-dinitrophenyl–conjugated antibodies as adaptor molecules to generate anti–gp160 CAR–NK cells that recognize various epitopes of HIV gp160 to mediate killing of target cells (137). As illustrated above, CAR–NK cells have numerous advantages that suggest they could be useful in future treatments for eliminating viral reservoirs.

## 7 Conclusion

**Figure 1:**
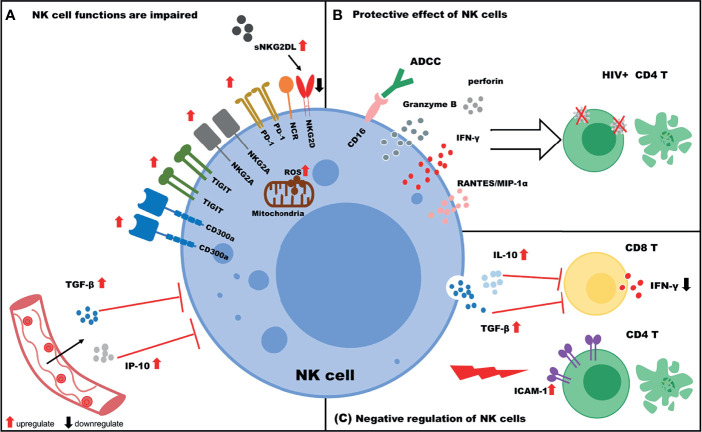
The role of NK cells in HIV infection. **(A)** NK cell functions are impaired. The expression of NK cell inhibitory receptors (TIGIT, PD-1, CD300a, and NKG2A) is upregulated, and the expression of activating receptors (NKG2D and NCR) is downregulated. Increased IP-10/TGF-β levels in plasma negatively regulate NK cell functions. In addition, increased levels of soluble NKG2DL (sNKG2DL) lead to downregulation of NKG2D expression on NK cells. **(B)** Protective effect of NK cells. NK cells target HIV+ CD4 T cells *via* ADCC or the secretion of various cytokines (IFN-γ, Granzyme B, perforin) and chemokines (RANTES, MIP-1α). **(C)** Negative regulation of NK cells. NK cells inhibit the function of CD8 T cells by secreting IL-10/TGF-β and killing uninfected CD4 T cells due to high expression of ICAM-1.

With respect to HIV infection, the functions of NK cells could be thought of as “two sides of the same coin”. The function of NK cells is impaired during HIV infection, and in some cases, the cells can exert negative regulatory effects on other immune cells. Nevertheless, NK cells still exert protective effects against HIV infection (**Figure 1**). The numbers and frequencies of various NK cell subpopulations change during HIV infection. In addition, an imbalance in the immune receptor distribution occurs, and the functions of NK cells are decreased. Furthermore, NK cells suppress the functions of T cells by secreting cytokines such as TGF-β and IL-10. In contrast, NK cells are still capable of lysing HIV-infected cells and suppressing viral reservoirs *via* the secretion of cytokines such as IFN-γ, and in this context, NK cells are negatively associated with progression of HIV infection. The functions of NK cells can be enhanced by treatment with inhibitory receptor inhibitors, LRAs, or by cytokine stimulation. Furthermore, CAR–NK cells could be beneficial for eliminating HIV reservoirs.

## Author Contributions

YS wrote the manuscript. JZ collected the data. YJ provided valuable guidance and revised the manuscript. All authors contributed to the article and approved the submitted version.

## Funding

This work is funded by National Natural Science Foundation of China (Grant Number: 82172341), and Scientific Research Funding Project of Higher Education in Liaoning province (Grant Number: LJKZ0737).

## Conflict of Interest

The authors declare that the research was conducted in the absence of any commercial or financial relationships that could be construed as a potential conflict of interest.

## Publisher’s Note

All claims expressed in this article are solely those of the authors and do not necessarily represent those of their affiliated organizations, or those of the publisher, the editors and the reviewers. Any product that may be evaluated in this article, or claim that may be made by its manufacturer, is not guaranteed or endorsed by the publisher.
